# A logic-based resilience metric for water resource recovery facilities

**DOI:** 10.1039/d4ew00649f

**Published:** 2024-10-31

**Authors:** Anna S. Laino, Ben Wooding, Sadegh Soudjani, Russell J. Davenport

**Affiliations:** a School of Engineering, Newcastle University Newcastle upon Tyne NE1 7RU UK a.s.laino2@newcastle.ac.uk; b School of Computing, Newcastle University Newcastle upon Tyne NE4 5TG UK; c Max Planck Institute for Software Systems Kaiserslautern Germany

## Abstract

This study develops quantifiable metrics to describe the resilience of Water Resource Recovery Facilities (WRRFs) under extreme stress events, including those posed by long-term challenges such as climate change and population growth. Resilience is the ability of the WRRFs to withstand adverse events while maintaining compliance or an operational level of service. Existing studies lack standardised resilience measurement methods. In this paper, we propose a resilience metric based on signal temporal logic (STL) to describe acceptable functionality of the WRRFs (*e.g.* meeting regulatory limits). By using Monte Carlo simulations and scenario optimisation on a model of a WRRF, we determine the maximum stress the WRRF can handle while meeting STL constraints for biochemical oxygen demand (BOD) and chemical oxygen demand (COD) compliance limits. The results are applied to a simple digital model of a facility with 22 components. Importantly, this method can be applied to data that water companies routinely and regularly monitor, and could be incorporated into SCADA systems. In our case studies, we determine threshold stressor values of extreme rainfall that result in a loss of resilience. Our results offer insights into the design of more resilient treatment processes to reduce environmental impacts.

## Abstract 2

A new notion of resilience using signal temporal logic from computer science.

## Graphical abstract

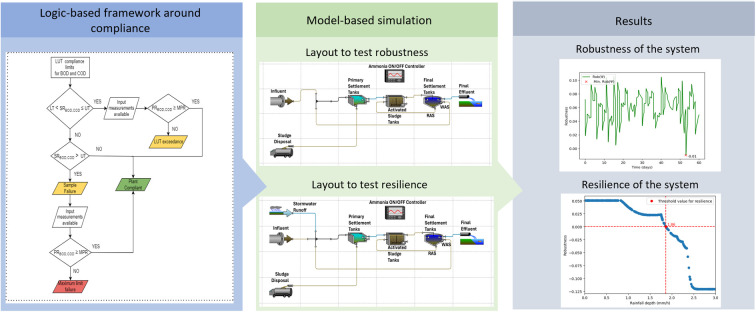

**Water impact**

The study addresses the absence of a general methodology for quantifying resilience in Water Recovery Resource Facilities (WRRFs). Signal temporal logic is introduced as an adaptable formalism, allowing easy adjustments to compliance regulations without altering the metric in its quantitative significance. The integration of STL specifications in real-time systems could improve WRRF monitoring, fostering resource recovery and safe water recycling.

## 1 Introduction

Critical infrastructures (CIs) such as power systems, water systems, telecommunications, and transportation networks play a vital role in sustaining modern societies and economies by providing essential goods and services for continuous functioning.^1,2^ The resilience of CIs is a comprehensive measure of their ability to withstand, respond to, recover from, and adapt to disasters.^3^ Numerous recent studies indicate that the assessment of resilience through indicators has become a common practice in managing CIs. The definition and characterisation of these indicators can bring benefits to society and industry in terms of safety, facilitating the monitoring and the enhancement of the capacities, and performance of CIs.^4^ Water Resource Recovery Facilities (WRRFs) are an important CI where the topics of resilience and its measurement have recently received attention. WRRFs are exposed to stressors that put pressure on the system, for example, anthropogenic activities that cause environmental pollution and/or extreme weather events including droughts or heavy rainfall.^5^ The number of serious pollution incidents in England's nine water and sewerage companies rose to 62 in 2021, the worst performance since 2013.^6^ Despite the serious pollution incidents reducing to 44 for 2022, the number of pollution incidents increased to 2026 in 2022 from 1883 in 2021.^7^ This coincided with England's hottest and wettest decade since records began (2012–2022) and an approximate 6% increase in UK population over the same time period.^8^ Processes in WRRFs that are resilient to these stressors provide greater reliability; enabling the recovery of more nutrients, energy, and other resources, while recycling water safely to the environment.

Butler *et al.* (2017)^9^ define a stressor (*a.k.a.* threat or disturbance) as any event which has the potential to reduce the degree to which a system delivers a defined level of service. In their work, they developed four threat subcategories: external-chronic, external-acute, internal-chronic and internal-acute. These categories lead to two classifications of threats: chronic stressors and acute stressors. Particularly, acute stressors are sudden and unpredictable.

The Intergovernmental Panel on Climate Change's (IPCC) Sixth Assessment Report (AR6) highlighted that climate change will increase the planet's average temperature by at least 1.5 °C within the next few decades compared to the pre-industrial levels during 1850–1900.^10^ Climate change is a critical challenge of this century and is classified as either an external-chronic threat or an external-acute threat. Due to climate change, WRRFs are expected to experience more severe stressors more frequently. Climate variability is expected to increase, causing both flooding and prolonged periods of dry weather. These can affect sedimentation dynamics in the sewerage systems and the occurrence of “first flush” pollutant loads.^5^ Another likely stressor for WRRFs is population growth, which is an external chronic threat. Indeed, the population of the United Kingdom (UK) is predicted to increase by 2.1 million by mid-2030, and is projected to reach 69.2 million over the next decade.^8^ However, Office for National 63 Statistics (2024)^11^ forecasted that the UK population might reach 70 million by mid-2026, a decade earlier than previous forecasts made in 2021. Population growth affects the resilience of water supply and WRRF systems, due to ensuing increases in flow rate (hydraulic overloading in the influent) and operational constraints (under performance of the process units in the system).^12^ WRRFs, whose system designs date back to the early 20th century, show a lack of resilience^13^ due to ageing infrastructure and their long design lifespans. In the case of unforeseen events such as equipment failures or extreme weather, these issues can be further exacerbated, causing the WRRFs to operate less efficiently and effectively, leading to compliance failures, and impacting the long-term reliability and resilience of such systems. Consequently, they may exhibit poor performance in terms of meeting compliance regulations. This impacts the long-term reliability of the facility, further exacerbating its lack of resilience. WRRFs may experience performance failures when operating outside the parameter ranges they were designed for, these include significant changes to the assumed flows, sewage characteristics, or climate conditions. Therefore, more frequent heavy rain or increases in temperature could significantly affect wastewater infrastructure. Higher rainfall intensity would increase flows through the water collection system, thereby conveying higher levels of pathogens to rivers and diluting organic and nutrient loads to WRRFs, which may compromise their biological processes. Low flows, triggered by drought, also cause issues in WRRFs, such as septicity in pipes and/or increased organic and nutrient concentrations. These events can impact the reliability and operating costs of WRRFs.^14^

Under future stressors, water supply and WRRF systems may not perform sufficiently to satisfy their service requirements. As a consequence, the environment may suffer serious pollution incidents due to a lack of compliance with treatment standards.^5^ Understanding how different WRRF processes respond to threats will play a fundamental role in adapting to climate change and an increasing population.^15^

### Research rationale

It is important to develop a general approach that compares the effects of stressors on WRRF resilience to understand present and future vulnerabilities. A generalised methodology to quantify and track resilience is not implemented by water companies since performance-based resilience metrics are either case specific or difficult to apply universally.

Sweetapple *et al.* (2022)^16^ described a general resilience assessment methodology (GRAM) that decomposes the general resilience of a water system through a middle-state based approach. GRAM takes into account the impact of any threat, whether known or unknown, on a system, provided that all possible failure modes of the system can be identified. For the application of this approach, it is not necessary to have a comprehensive knowledge of the stressors affecting the system. Our approach aligns with the GRAM methodology; however, the currently used performance-based levels of service advocated by Sweetapple *et al.* (2022),^16^ are not based on regulatory water quality standards. Furthermore, stress/failure modes are arbitrarily quantified and are not necessarily related to the quantities monitored by water companies (dissolved oxygen and un-ionised ammonia concentrations). Our approach could facilitate the quantitative comparison and analysis of stressors to better understand how to increase the resilience of WRRFs that contribute to increasing the resilience in a WRRF. For instance, it allows for the identification of the maximum threshold value of a stressor (or multiple stressors) at which the WRRF can still comply with regulations. This study aims to introduce a new framework and metric for quantifying resilience as a proof of concept that could be incorporated into GRAM and offer further insights for water companies into managing their WRRFs.

We propose for the first time a new strategy and metric with which to quantify resilience founded on temporal logic reasoning that captures the compliance requirements and incorporates a measure of how long a WRRF can recover, adapt or fail in relation to regulatory water quality standards.

## 2 Resilience review

### 2.1 Resilience background

The concept of resilience has been applied to various fields of study and in numerous contexts, including ecology, economics, and psychology. In engineering is has been used to help plan and design urban infrastructures. Scientists and engineers define resilience with multiple subtly different definitions but with shared similarities.^17^ A precise definition and quantification are therefore challenging.

Holling (1973)^18^ was a pioneer of the resilience concept. His qualitative resilience definition was based on the adaptive capacity of an ecological system. In his definition, an ecological resilient system was considered to be able, under dynamic conditions, to absorb disturbances or shocks, and to change a previously stable state into a new stable one. This was possible by changing a system's structure while maintaining its functionalities. DeAngelis (1980)^19^ also investigated resilience for ecosystems and he defined resilience as “the speed with which a system returns to equilibrium state following a perturbation”. After the work of Holling (1973),^18^ successive research has focused on developing resilience metrics for various fields.

Considering ecosystems, Holling (1973)^18^ explained that the development of resilience metric(s) would require deep and comprehensive knowledge about ecological systems, which was often difficult to attain. Resilience in the context of engineering systems took on a new meaning after further developments by Holling (1996).^20^ The design of engineering systems are expected to provide reliability, the capability to swiftly cope with disturbances and to ensure rapid recovery to normal operating conditions. However, achieving all these aspects is not always feasible due to various factors. Older systems may lack redundancy in their equipment, and a shortage of funding to improve facility operations, including investments in well-trained personnel. The inherent complexity of modern engineering systems can also pose challenges to implementing robust resilience measures. Furthermore, while engineering systems endeavour to cope with most disturbances, the severity and nature of certain events may result in prolonged recovery times, despite best efforts. Therefore, while the aspiration is for engineering systems to rapidly recover from disturbances, achieving this goal may not be universally attainable in practice. What emerges from Holling's work is that the distinction between engineering systems and ecological systems: engineering systems require human intervention to return to their original steady state after a perturbation occurs. WRRFs employing biological processes, are therefore considered hybrid systems as their behaviour is somewhere between ecological and engineering systems. Various definitions of resilience for WRRFs can be found in the literature, but there is no universal resilience metric (qualitative and/or quantitative) that can be applied across all scenarios.

### 2.2 Resilience properties of urban water systems

Resilience is a vital concept in urban water management. Recent studies in such systems have focused on identifying the main characteristics of a resilient system, these include; robustness, adaptability, resourcefulness, reliability, and speed of recovery. These properties aid urban water management systems to resist, cope with, and adapt more quickly to stressors. Furthermore, these properties should be recognised as resilience indicators and must be quantified either qualitatively or quantitatively through metrics. The speed of recovery is an important parameter for a resilient system. It is considered as the time that the system takes to return to its performance levels before the stressor was applied.

Reliability is associated to the probability of successful operation of the system,^21^ or equally the probability of being in a non-failed state.^22^

Niku *et al.* (1979)^23^ defined reliability as “the ability to perform the specified requirements free from failure” or “the probability of adequate performance for at least a specified period of time under specified conditions”. In their paper, the authors analysed the concentrations of BOD and the suspended solids (SS) in 37 WRRFs to determine a probabilistic model to predict achievable concentrations for BOD and SS. Butler *et al.* (2014)^22^ developed the Safe and SuRe framework for urban water management, stating that systems in this century must be safe, synonymous with reliable, and also resilient, with a strong link to sustainability. They defined resilience as the “degree to which the system minimises level of service failure magnitude and duration over its design life when subject to exceptional conditions”. In this definition, resilience is associated with the performance response of a system following an unexpected event which might lead it to fail the designed level of service. These authors recognised the lack of a general method, and therefore further developed and improved this framework as a set of guidelines.^9^ Resilience has been generally and simply defined as the capacity of a system to “bounce back”.^24–26^

Sweetapple *et al.* (2019)^27^ analysed the link between resilience and sustainability; where increases in resilience may provide improvements in sustainability. Sustainability is a normative concept, referring to physical and institutional practices which meet the needs of the present without compromising the ability of future generations to meet their needs.

### 2.3 Resilience metrics in urban water systems

In CI, resilience also has further wider meaning and implications in relation to vulnerability to disasters and the interdependence of systems. This is out of scope of the current study, but resilience metrics for such scenarios have been a developed a good example of which is given by Jia *et al.* (2023).^28^ The authors developed a new two-stage stochastic optimisation model to determine both the locations for building restoration team stations before disasters and their routing for conducting restoration tasks after disasters simultaneously. Implementing pre-disaster planning and post-disaster scheduling are typical strategies aimed at enhancing the resilience of CI. In the literature pre-disaster measures include selecting resilient facility locations, building relief centres, and protecting critical components. Post-disaster efforts involve restoring services, comparing recovery strategies, and optimising restoration processes. Some studies consider both pre- and post-disaster optimisation, while others explore protecting critical components before disaster strikes. Hosseini *et al.* (2016)^24^ argued that a resilience metric without an accompanying framework is limited, as it lacks the necessary guidance for practical implementation. In the context of urban water systems, resilience metrics are typically developed following a risk assessment of a specific case study, examining particular scenarios and stressors. Risk analysis complements resilience efforts, even though it may not encompass unknown threats.^9^ It is crucial to distinguish between methods aimed at mitigating risk and those focused on enhancing resilience. While risk-oriented approaches assist in preparing for events with familiar patterns, resilience-oriented approaches are aimed towards empowering systems to effectively respond to any eventuality, including unforeseen and unprecedented circumstances.^29^ Indeed, risk assessments are necessary to forecast undesired unexpected events and aim to mitigate negative effects on a system. Resilience is associated with intrinsic characteristics of a system that make it able to cope with undesired events. Resilience is a key parameter when the risk of unwanted events happening cannot be computed.^30,31^

The work by Francis and Bekera (2014)^32^ provides a resilience assessment framework, which was the first to include the engagement of the stakeholders and a metric for evaluating resilience under deep uncertainty. The resilience metrics are performance based and take into account the speed of recovery after a performance loss. Another approach for quantitative metrics is to consider the performance of the wastewater system under multiple threats. Example resilience metrics include: the efficiency of removal of pollutant concentrations in final effluent, the speed of recovery of the system after a disruption, or the reliability of the system.

Weirich *et al.* (2015)^33^ used a general linear model for *post hoc* statistical analysis of performance, resilience, and stability of secondary WRRFs over 41 months. They demonstrated that WRRFs which failed in the past had a statistically increased likelihood of failing again. In the literature, resilience metrics are associated to a risk analysis for a specific scenario^30^ and often on specific unit processes within a system. By considering a system, all possible stressors, and their probability of occurrence, the resilience metric will contain all the parameters that play a role in affecting resilience. In literature, resilience has been described through the change in performance or function over time. Cuppens *et al.* (2012)^34^ addressed resilience as a performance indicator for a system under disturbance. The authors highlighted the importance of simulating a dynamic disturbance for better analysis. Similarly, Mugume *et al.* (2014)^35^ focused on quantifying resilience for urban drainage systems for flooding. In particular, their resilience metric assesses the remaining functionality of the system at different levels of link failure by combining both the failure's scale and duration into a single measurement. Following their previous study, Mugume *et al.* (2015)^36^ applied and extended the global resilience analysis (GRA) methodology to a urban drainage system measuring a new resilience index combining the failure magnitude and the duration. GRA considers the system performance when it is under various stressors. Using a case study, they developed a metric to quantify the system residual functionality under various failure scenarios. The resilience index connects the resulting loss of functionality to the system's remaining functionality, which indicates the level of resilience at various levels of link failure. The authors define the severity as the reduction in system functionality. Severity is characterised by the highest degree of failure magnitude (peak severity) and the duration of the failure.

Holloway *et al.* (2021)^13^ defined “dynamic resilience” of the biological components in a WRRF. They decoupled stressor events (cause) from process stress (effect) to track the system deviation from normal conditions. The authors used Monte Carlo simulations to compute the probability of failure and then scaled the outputs to show, using a traffic light system, where the biological system stands under certain conditions of stress. This approach shows potential, but success for implementation on other WRRFs requires a large number of samples and data.

One method for broadly evaluating resilience in water systems is failure modes and effects analysis (FMEA). This is a proactive method to identify potential failure modes in a system, and it can help discriminate between them, ranking the severity of each failure, or help discriminate the probability of the occurrence of these various failures. Similar to the FMEA approach,^37^ GRAM is beneficial in identifying system failure modes, and to plan interventions to make the system more resilient to unforeseen threats in a quantifiable way. For the application of this approach, it is not necessary to have a comprehensive knowledge of the stressors affecting the system.

Xue *et al.* (2015)^38^ posed resilience as the core evaluation of a sustainable system and highlighted non-standardisation of resilience assessments/metrics. The resilience assessment highlighted the importance of focusing on the future changes and challenges that can affect the correct operation of the WRRFs. Similarly, Schoen *et al.* (2015)^39^ defined resilience as the “ability to prepare for and adapt to changing conditions and withstand and recover rapidly from disruption”. Furthermore, Cuppens *et al.* (2012)^34^ defined robustness as the ability of a WRRF to withstand a disturbance without decreasing the performance.

In this paper, we consider the notion of robustness as how close the system is to compliance failure under normal operations. Robustness is commonly mistaken for resilience, and is a measure of the strength of a system. Whereas, resilience is a measure of the flexibility, adaptability, and agility of a system to withstand a stressor without failing the compliance limits, or to recover quickly after a compliance failure. Additionally, resilience is enclosed in the system's operation through controls, while robustness is a property which is embedded in the system's design.^31^

## 3 Methodological approach

**Fig. 1:**
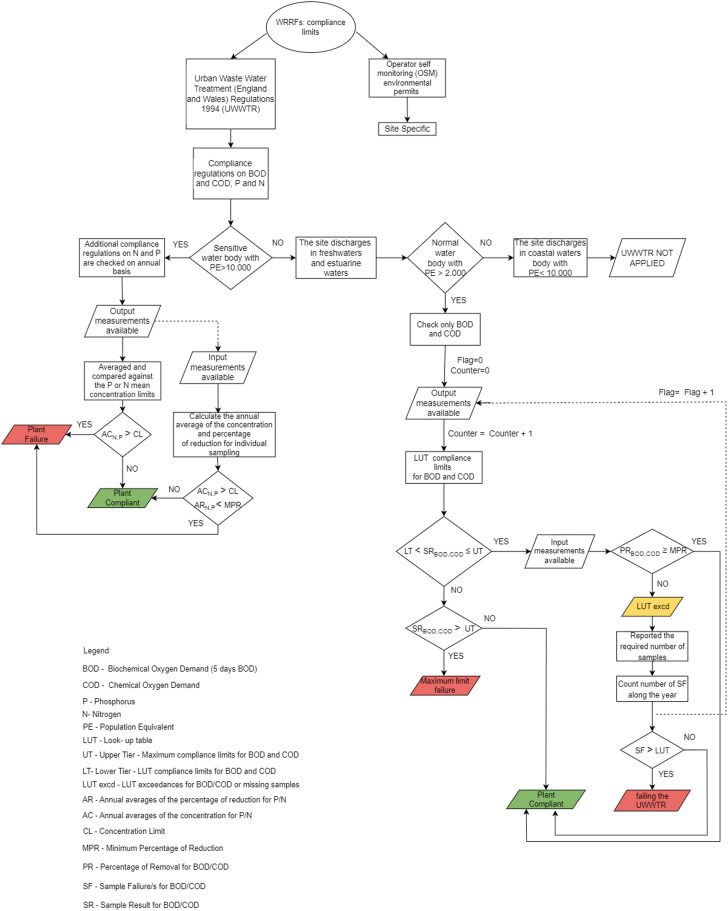
The logic diagram for the compliance regulations on WRRFs.

In this paper, we introduce a logical framework (*cf.*Fig. 1) and a case study implementing the framework using data and simulations of a WRRF in Scotland under the management of Scottish Water. Our methodology consists of two main parts. The first part defines an STL specification for robustness that considers compliance. This is a widely applicable approach and can be used as a screening tool to determine the robustness of WRRFs. The second part involves a detailed analysis of resilience using the software tool GPS-X Hatch^40^ simulator to test the particular stress scenario of a rainstorm. The use of GPS-X aids in measuring the intrinsic robustness of the facility, the methodology can be build upon scenario testing to understand the factors that affect resilience. The aim of measuring robustness is to understand system operations under normal operation conditions. We want to see how much we can push the system to observe compliance failures. In addition, robustness and resilience metrics together can be used to determine stressor threshold limits, beyond which a given WRRF is likely to become non-resilient.

### 3.1 UK compliance regulations

Interviews with process scientists working for Scottish Water and Northumbrian Water Ltd highlighted compliance limits as the main threshold(s) where a lack of resilience can be detected. It is first necessary to establish, in its current configuration and operational rule sets, the robustness of a treatment asset, system, or process. We will develop a logical framework, based on compliance regulations for the UK, to provide a robustness value for any given WRRF.

Water quality monitoring in the UK has been governed by regulatory bodies such as the Environment Agency in England, Scottish Environment Protection Agency in Scotland, Natural Resources Body for Wales, and the Department of Agriculture, Environment, and Rural Affairs in Northern Ireland.

The regulations behind compliance limits are an intricate system divided in two main parts: common regulations for sites with a population equivalent or greater than 2000, and site-specific regulations for a given WRRF. The WRRFs must be compliant under Urban Waste Water Treatment (England and Wales) Regulations 1994 (UWWTR), which implements the European Union Urban Waste Water Treatment Directive (91/271/EEC), and the operator self-monitoring (OSM) environmental permits. Fig. 1 shows the compliance regulations as a logic diagram. It shows a complete flowchart for UWWTR which are the compliance constraints that are not site specific but apply to all sites with a population of 2000 or greater. Furthermore, it shows the different levels of failure for a given parameter.

### 3.2 Resilience logical framework using STL

STL is a specification language which can be used to express properties of timed signals for real-time systems.^41,42^ The implementation of these logical statements enable checking the satisfaction of a property *via* a binary true/false representation.^43^ It has the advantage of admitting quantitative semantics which we refer to as a robustness function (see Appendix B). It provides a logic-based structure used to describe acceptable behaviour of reactive systems. STL is particularly useful when specifying properties of dense-time real-valued signals.^44^

**Fig. 2:**
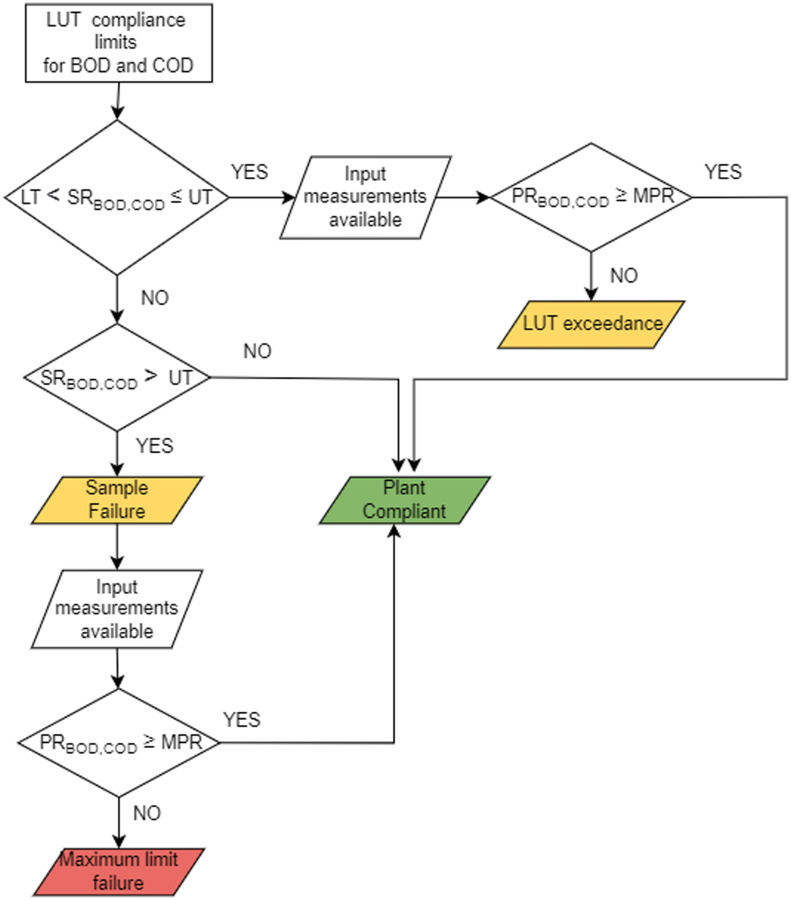
Subset of the logic diagram for the compliance regulations on WRRFs.

The compliance regulations of UWWTR for a WRRF put restrictions on BOD, COD, nitrogen (N) and phosphorus (P). In the following we will analyse COD and BOD, while N and P will be addressed in future work. When the WRRF is under the influence of stressors, it is expected to return to normal operation eventually. Normal operation is judged as satisfaction of the compliance requirements set by the UWWTR, see Fig. 1. In this first development of this framework, we consider only a subset of the logic diagram for the compliance regulations on WRRFs as presented in Fig. 2.

Firstly, we will write the compliance constraints as logical statements. A logical statement can be true or false. It is constructed as a hypothesis which has a precondition followed by a conclusion, where the conclusion is the key part to infer if the hypothesis is true. The following logical statements have been written using the threshold values from the look-up table compliance limits for BOD and COD^45^ following the guidelines of the UWWTR. The logical statements to check compliance against the UWWTR in Fig. 2 over time are:

• BOD concentration under the lower tier BOD_LT_ = 25 mg l^−1^ O_2_ or the minimum percentage of reduction BOD_%_ must above 70%;††For some facilities this could be up to 90%.

• BOD concentration always under the upper tier BOD_UT_ = 50 mg l^−1^ O_2_;

• COD concentration under the lower tier COD_LT_ = 125 mg l^−1^ O_2_ or the minimum percentage of reduction COD_%_ must be above 75%;

• COD concentration always under the COD upper tier COD_UT_ = 250 mg l^−1^ O_2_.

We now write these logical statements as STL formulae. The definition and syntax of STL formulae can be found in Appendix A and Appendix B. We denote BOD influent concentrations as *x*^1^_*i*_(*t*), COD influent concentrations as *x*^2^_*i*_(*t*), BOD effluent concentrations as *y*^1^_*i*_(*t*), and COD effluent concentrations as *y*^2^_*i*_(*t*). The concentrations are change over time *t*, and *i* is the index for the concentration, which changes in some range [0, *n*] where *n* ∈ ℕ, and ℕ is the set of natural numbers including zero.

Furthermore, the STL specification of the compliance regulations for BOD and COD is denoted by *ψ*. The symbol 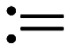 is read as “is defined to be equal to”. Subscript % denotes the minimum percentage of reduction. The symbol □ is a temporal operator used in STL to mean “always”. The logical operators ∧ and 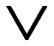 mean respectively “and” and “or”, and [*a*, *b*] is the interval of time considered for the simulation.
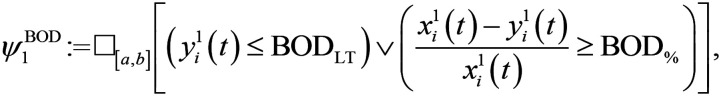

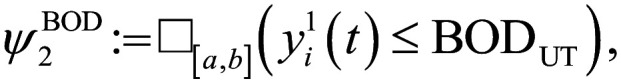
*ψ*^BOD^ = *ψ*^BOD^_1_ ∧ *ψ*^BOD^_2_.and
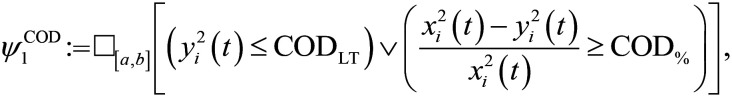

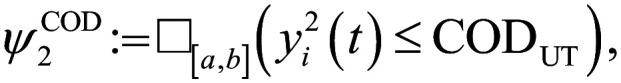
*ψ*^COD^ = *ψ*^COD^_1_ ∧ *ψ*^COD^_2_.

The major advantage of the STL formalism is its adaptability. The specifications can be easily changed if the compliance regulations change, *e.g.* the thresholds for the upper or lower tier or the percentage of reduction values, yet the metric and its quantitative significance would remain unchanged. The behaviour of the system can then be checked against the STL specification to see if the system is operating as expected and how close the system is to failure. In this study, satisfaction of the STL specification represents the satisfaction of regulatory requirements and other expected recovery behaviour under stressors. The STL formula used for the specification defines how resilient the system is, at any point in time against any given stressor or multiple stressors. In this study, we introduce a comprehensive framework for water companies, encompassing various applications. This framework enhances the resilience monitoring of WRRFs by refining compliance assessments through routine data checks, including parameters such as BOD and COD. Notably, our approach involves running continuous dynamic simulations in GPS-X Hatch with a specific time step, allowing for a comprehensive evaluation of overall system robustness. Importantly, it is worth noting that our framework can be applied equally to both continuous and composite data, with the different approaches not impacting the validity of the framework.

### 3.3 Model-based simulations

GPS-X Hatch is a globally available software tool for the design and operation of WRRFs. GPS-X Hatch is a mass balance based software tool which is used by modellers to simulate mass and energy flows within a WRRF. It has been used to build the model of a large WRRF in Scotland under Scottish Water management, used as the case study. This mechanistic model has been used for the verification of the logical framework based on STL specifications.

The WRRF serves a population equivalent to 574 000 and can treat a capacity of flow-to-full treatment (FFT) of 7.59 m^3^ s^−1^. It is an activated sludge plant (ASP) and discharges final effluent to an estuary. Scottish Water provided the data used to calibrate and validate the model following the IWA good modelling practice (GMP) protocol.^46^ The calibration of the mechanistic model was carried out over the period November and December 2021 (60 days of dynamic simulation). We identified a period where the WRRF was working under stable conditions. A parameter that we used to determine stable operation over the year was the MLSS (mixed liquor suspended solids).

Firstly, we performed a steady state calibration followed by a dynamic simulation to verify the fit with real data. Although the calibration of a real plant is important for referencing to a real world application, the accuracy involves many variables. Our framework maintains its conceptual integrity regardless of the specific data it encounters. We provide detailed information about the calibration of the COD effluent in the Appendix C.

We used the stress–strain methodology which was developed in solid mechanics to study the behaviour (strain) of solid materials under a load (stress). The stressors are applied, with varying the magnitude and the duration, to establish a range of strain profiles.

We use GPS-X to test resilience scenarios by introducing stressors, especially for random and unexpected events, into the model and analysing the model strain outputs. The strain is linked with the final effluent concentrations to verify if the WRRF is compliant for a given scenario. Resilience is quantified by metrics that track the baseline position of the concentrations and report them against set targets over time.

### 3.4 Monte Carlo simulations

We stress the system by applying a rectangular design storm^47^ from the 23rd day until the 28th day of a 60 days simulation. A Monte Carlo simulation with 1000 sample outputs were computed using the Python tool in GPS-X Hatch. In computer science, Monte-Carlo algorithms are based on randomness to compute a large number of simulations on different scenarios. The outputs represent a variety of scenarios with different probability of occurrence. In our implementation of the Monte Carlo simulations, the rainfall intensity (mm h^−1^) is randomly picked between 0 and 10 at each simulation on a fixed length of the duration. The rainfall intensities are sampled from a uniform distribution for a rectangular design storm.^47^ Our computations can be extended to include random duration and random starting times as considered in ref. 48.

Note that the Monte Carlo simulation is used to provide an approximate solution for the optimisation in the definition of resilience. There is no error attached to these computations, and we only have convergence results: when the number of simulations goes to infinity, the computed value will converge to the optimal value. We have done 1000 simulations and performed curve fitting to get the approximate solution for the optimisation (*cf.*Fig. 10).

## 4 Results and discussion

### 4.1 Model-based simulation

**Fig. 3:**
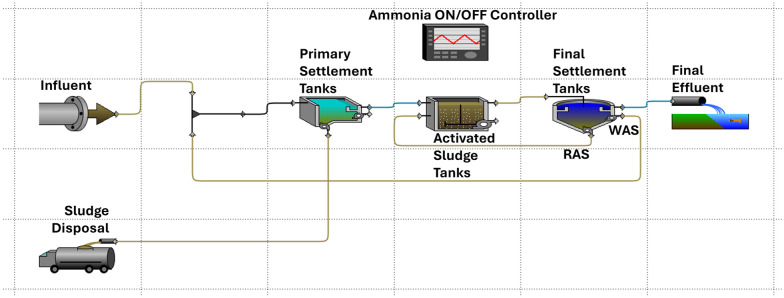
Layout of the WRRF case study before applying a stressor.

The model of the case study in GPS-X is showed in Fig. 3, where the system is presented in its design configuration. This layout has been used to compute the robustness of the system.

After computing the robustness of the system, we applied a stressor to test resilience and determine the maximum magnitude at which the system returns to normal operation.

**Fig. 4:**
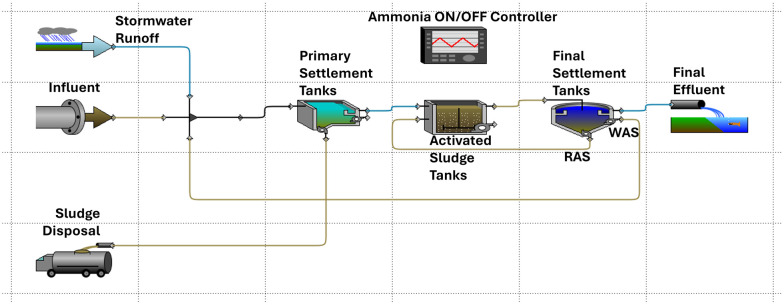
Layout of the WRRF case study after applying a stressor.

Fig. 4 shows the layout of the WRRF after applying a stressor.

### 4.2 Robustness

An STL formula or specification can be designed to provide a definition of space robustness or time robustness.^41^ Let us consider the effluent concentrations *y*_*i*_(*t*) where *i* ∈ [0, *n*]. The compliance regulations are then expressed by the STL formula *ψ* on these effluent concentrations.

#### Definition 1 (robustness)

The robustness of a WRRF is the maximum value *c* that can be reduced from *y*_*i*_(*t*) such that the plant still satisfies the compliance specification *ψ* at all time instances. The symbol 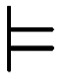 in *y*
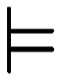
*ψ* is read as “entails”, and is used to show satisfaction of *ψ* by *y*. Therefore, robustness is:
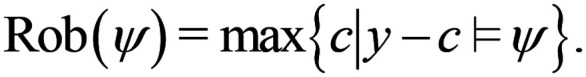
with *y* − *c* = (*y*_0_(*t*) − *c*, *y*_1_(*t*) − *c*, …, *y*_*n*_(*t*) – *c*).

The robustness Rob(*ψ*) can be computed recursively using the structure of *ψ* and the definitions in Appendix B. We have that if *y*
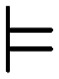
*ψ* then Rob(*ψ*) ≥ 0.

#### Remark 1

The above definition adds *c* to all outputs *y*_0_(*t*), *y*_1_(*t*), …, *y*_*n*_(*t*). It does not take into account that different outputs may have different ranges of values. For a given WRRF, we can normalise the outputs and map them into the same range [0, 1]. Let us consider linear mappings *f*_*i*_: ℝ → [0, 1] that shift and scale the outputs *z*_*i*_(*t*) = *f*_*i*_(*y*_*i*_(*t*)), *i* = 0, 1, …, *n*, such that *z*_*i*_(*t*) ∈ [0, 1]. Then we modify the definition of robustness as:1
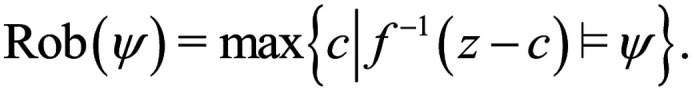
where *f* = [*f*_0_, *f*_1_, …, *f*_*n*_] and *f*^−1^ is the inverse function of *f*.

The robustness definition for the subset of the logic diagram in Fig. 2 has been implemented in GPS-X using:

2 *c*_BOD_ = min[(BOD_UT_ − *y*^1^_*i*_(*t*)), max[(BOD_LT_ − *y*^1^_*i*_(*t*)), −(BOD_%_ × *x*^1^_*i*_(*t*)) + *x*^1^_*i*_(*t*) − *y*^1^_*i*_(*t*)]].

3 *c*_COD_ = min[(COD_UT_ − *y*^2^_*i*_(*t*)), max[(COD_LT_ − *y*^2^_*i*_(*t*)), −(COD_%_ × *x*^2^_*i*_(*t*)) + *x*^2^_*i*_(*t*) − *y*^2^_*i*_(*t*)]].

**Fig. 5:**
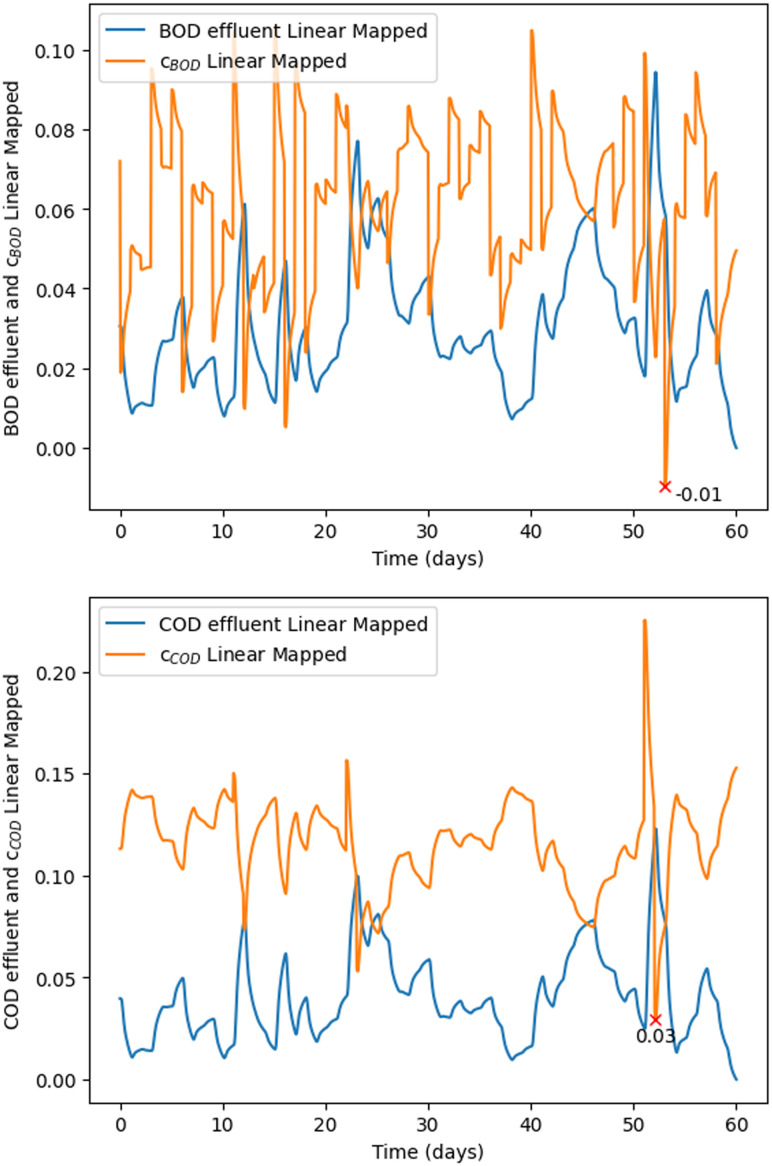
In both graphs, red “×” denotes the time and the magnitude when the systems was closest to failure. Top. Effluent concentrations (BOD) and robustness (*c*_BOD_) after linear mapping over a 60 day time horizon. Bottom. Effluent concentrations (COD) and robustness (*c*_COD_) after linear mapping over a 60 day time horizon.

By using a linear mapping on BOD and COD, we can compare and quantify the robustness Rob(*ψ*) of the system directly over the same range [0, 1]. The mapping *f*_*i*_ takes the following form:


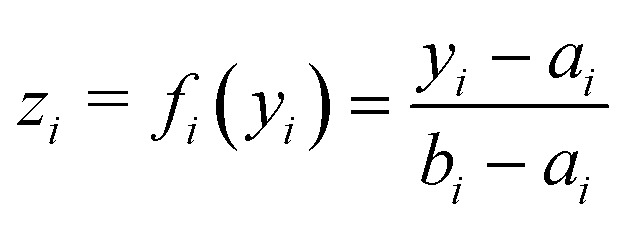
for any *y*_*i*_ ∈ [*a*_*i*_, *b*_*i*_], *e.g.* for BOD ∈ [25, 50] and COD ∈ [125, 250]. Fig. 5 shows BOD and COD effluent concentrations and the implementations of Eqn (2) and (3) after the linear mapping is performed.

Eqn (1) can also be written as:

4 Rob(*ψ*) = Rob{*c*_BOD_ ∧ *c*_COD_} = min[*c*_BOD_(*t*), *c*_COD_(*t*)].

which represents the robustness of the STL formula, see Appendix B. Eqn (4) (metric) shows when the WRRF is close to failing the compliance requirements after considering both COD and BOD.

**Fig. 6:**
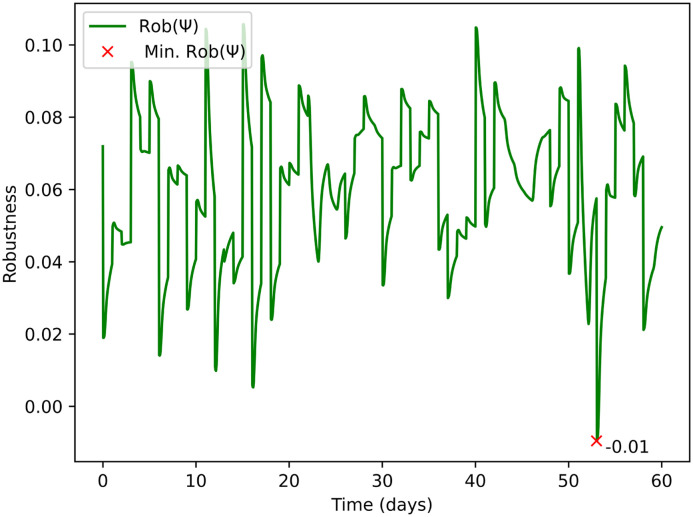
Robustness Rob(*ψ*) of the system across a finite-time horizon of 60 days, showing how close the system is to fail the compliance requirements. The red “×” denotes the day and the Rob(*ψ*) value when the system was closest to failure.

Rob(*ψ*) represents the robustness of the system considering the analysis on both BOD and COD, see Fig. 6. Eqn (2) and (3) within the framework's mathematical structure indicate when the system comes close to failing the required BOD and COD standards for the UWWTR. Rob(*ψ*) is therefore dimensionless. A lower Rob(*ψ*) denotes that the system is close to the threshold values of the compliance regulations. A negative value means that the system has already passed the compliance threshold, and consequently we can assume that the system is not working under normal design operation.

**Fig. 7:**
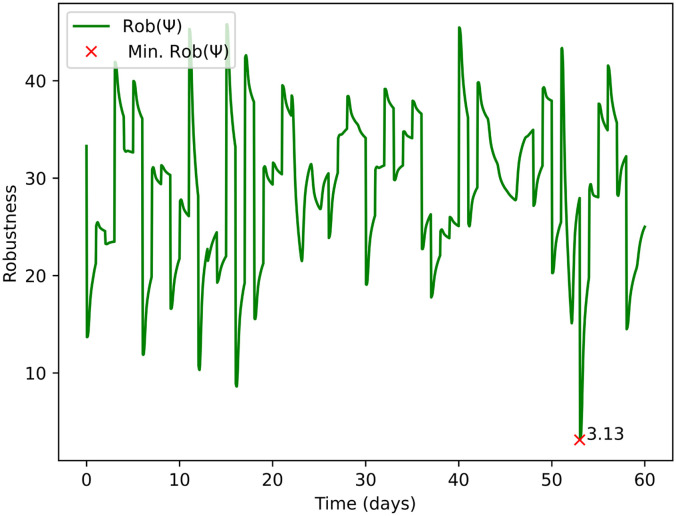
Robustness Rob(*ψ*) of the system across a finite-time horizon of 60 days after applying the inverse transformation of the linear mapping, showing how close the system is to fail the compliance requirements for the value of BOD. The red “×” denotes the day and the Rob(*ψ*) when the system was closest to failure.

After identifying Rob(*ψ*), following the structure in Appendix B for the STL specifications, we applied an inverse transformation to revert the changes. This will allow us to have a quantification of the parameter *c* in Eqn (1), as shown in Fig. 7. In Fig. 7 the red marker “×” indicates the day when the lowest Rob(*ψ*) occurs. The magnitude showed next to the marker “×” is meaningful value for quantifying the robustness of the plant. It could be used by water companies to rank their WRRFs, including prioritising them for interventions to avoid compliance failures.

#### Remark 2

The STL specifications scale well with the number of variables considered, beyond BOD and COD in this work. With two parameters it is easy enough to manually see how close the system is to failure but as more variables are included this becomes more challenging to do manually and this robustness metric becomes more valuable for failure detection.

#### Remark 3

Quantifying the exact parameter that causes failure may be more challenging for more complex problems. In particular, when specific combinations of parameters are the cause of failures. This robustness metric is valuable to detect the closeness of those complex failures, but it may still be challenging to diagnose which parameter or combination thereof is the root cause.

### 4.3 Resilience

#### Definition 2 (resilience)

Consider stressors *u* = [*u*_0_(*t*), *u*_1_(*t*), …, *u*_*n*_(*t*)] affecting the WRRF, *ψ* being the compliance specification, and another STL specification *ϕ* denoting the requirements on recovery from stressors. We define resilience of a WRRF to be the maximum stressors *u* = [*u*_0_(*t*), *u*_1_(*t*), …, *u*_*n*_(*t*)] that can be applied to the WRRF while the effluent concentrations *y* = [*y*_0_(*t*), *y*_1_(*t*), …, *y*_*n*_(*t*)] and the influent concentrations *x* = [*x*_0_(*t*), *x*_1_(*t*), …, *x*_*n*_(*t*)] still satisfy both specifications *ψ* and *ϕ*:5
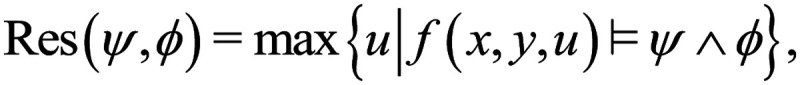
where *f*(*x*, *y*, *u*) is the function which has as variables the influent *x* and effluent *y* concentrations and the stressor *u*.

Example of the specification *ϕ* includes the following: if the effluent concentrations *y* under the stressors go above a certain threshold *y*_rec_, then *y* should go below this threshold within time interval [0, *T*]. This is denoted by the specification
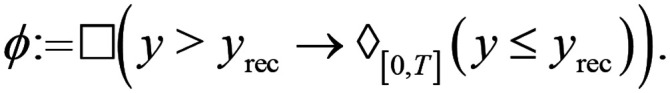
Another example for *ϕ* is the recovery requirement that the system go back to normal operation within time interval [0, *T*] once the compliance requirements have been violated 
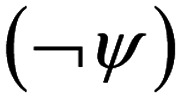
:6
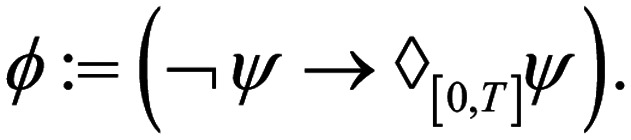
The STL operator ⋄_[0,*T*]_ means the system will eventually satisfy the compliance requirements within the range of time *T*, see Appendix A. *T* could be in the order of days (*e.g.* during winter due to the dilution) or the order of hours (*e.g.* in summer, when the weather is warmer, the final effluent is less diluted and has a higher concentration of pollutants).

Res(*ψ*, *ϕ*) can be used to determine the maximum threshold stressor value that ensures the effluent concentrations still meet the compliance and recovery requirements.

#### Remark 4

Our definition of resilience is not restricted to the choice of the time horizon *T* or the specifications *ψ* and *ϕ*. The provided definition can be applied to any compliance specification *ψ* and any specification *ϕ* that expresses recovering to a normal operation under the stressors.

#### Remark 5

The characteristics of our definition of resilience are not captured in the traditional definition of risk and safety. Safety measures try to prevent the system from failures while our definition of resilience captures violation of compliance requirements under stressors and the capabilities of the system to recover from such stressors. Our definition does not include any notion of risk of failures but measures the magnitude of stressors that the system can tolerate while being compliant and recover from the stressors. Reliability engineering in a safety-I perspective defines safety as a condition where the number of adverse outcomes is as low as possible.^49^ Instead, we propose to define “how performance go well under stressors” using temporal logic specifications, then consider a system to be more resilient than another if it has the same good performance under larger stressors. This is captured in our Definition 2 by including the expected behaviour of the system recovering from stressors as the temporal logic specification *ϕ*.

#### Remark 6

The function *f* in Eqn (5) captures the dynamics of the system that maps the stressors and other inputs to the outputs of the system. This will give a resilience metric specific to each system.

**Fig. 8:**
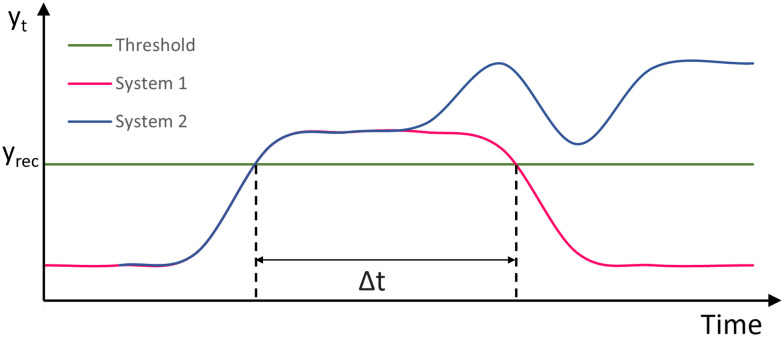
Examples of responses of two systems under a stressor. System 1 (magenta) is resilient recovering within time Δ*t* ≤ *T*. System 2 (blue) is not resilient as it does not recover at any time.

**Fig. 9:**
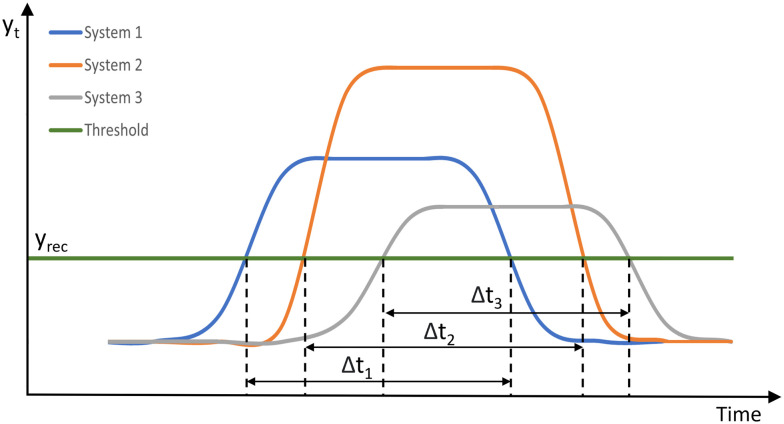
Recovery behaviours of three systems under stressors. Systems exhibit acceptable recovery behaviours when the times Δ*t*_1_, Δ*t*_2_, Δ*t*_3_ ≤ *T*.

Fig. 8 shows two systems under the same stressor. System 1 is resilient as it recovers from the stressor when Δ*t* ≤ *T*, while system 2 is not resilient since its output does not fall below the threshold line *y*_rec_. Our definition changes the perspective by specifying the set of acceptable recovery behaviours by *ϕ* as *e.g.* in Eqn (6) and then comparing different systems with respect to the maximum stressor they can tolerate while showing an acceptable recovery behaviour. Fig. 9 illustrates the recovery behaviours of three systems, which are acceptable according to *ϕ* if the times Δ*t*_1_, Δ*t*_2_, Δ*t*_3_ are less than the specified threshold *T*. System 1 (blue) recovers within Δ*t*_1_, system 2 (orange) recovers within Δ*t*_2_, and system 3 (grey) recovers within Δ*t*_3_.

The optimisation in Eqn (5) for the computation of resilience becomes a multi-objective optimisation when the set of stressors has more than one parameter. In the next section, we discuss how to do the computation when the set of stressors can be characterised with only one parameter.

### 4.4 Monte Carlo simulations

**Fig. 10:**
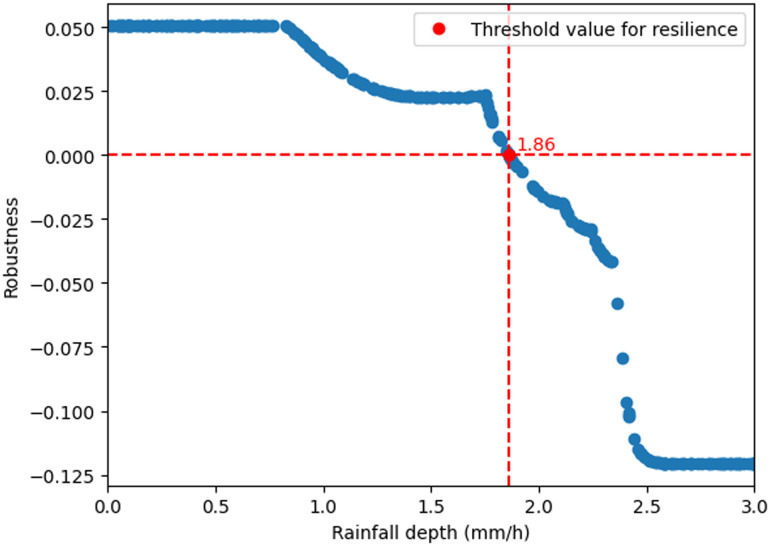
Robustness of the system under rainfall computed for different rainfall intensities (mm h^−1^). The stressor is applied to the inlet of the system. The resilience (red dot) is the largest rain intensity while still having non-negative robustness. This is equal to 1.86 mm h^−1^.

We stressed the system by applying a rectangular design storm from the 23rd day until the 28th day of a 60 days simulation. A Monte Carlo simulation with 1000 sample outputs were computed using the Python tool in GPS-X Hatch. The python code that performed the simulations picked the values from a uniform distribution. A subset of the simulation outputs that show the resilience of the WRRF case study under a stressor are presented in Fig. 10.

The rainfall intensity (mm h^−1^) threshold value to apply at the inlet for having Res(*ψ*, *ϕ*) = 0 is approximately 1.86 mm h^−1^. For values above this threshold, the system starts to fail the compliance regulations. This threshold is the resilience metric defined in Eqn (5). Given the critical failure threshold at 1.86 mm h^−1^ and the testing range extending from 0 to 10 mm h^−1^, it appears that the system exhibits limited resilience, particularly in light of the observed challenges at the lower end of the spectrum.

We performed an analysis of rainfall data for the November–December 2021 period. The data revealed notable peaks reaching up to 25 mm d^−1^ with average daily rainfall during this period being 2.19 mm d^−1^. Since our resilience metric is targeting the behaviour of the system under extreme events, multiple simulations should be obtained under different stressors to find a suitable range for rainfall densities that make the system violate the compliance (and potentially other recovery) requirements. This range is not necessarily associated with datasets that contains data points being observed under normal circumstances, but it is associated with data points that are rare and can be observed with very small probability (*i.e.*, extreme events that has happened a few times in the life cycle of the system^50^). In our model, the system exhibited signs of violating compliance requirement beyond a threshold of 1.86 mm h^−1^. Given the observed limitations of the system and the desire to understand its behaviours under more extreme conditions, we opted to push the simulation by introducing higher rainfall intensities. This deliberate choice aims to stress-test the system and compute our resilience metric.

## 5 Conclusions

This paper demonstrated, for the first time, development of a logical framework using STL specifications that provide the basis of a general method allowing water companies to track the robustness and resilience of their facilities. This framework can also help the water companies to enact better management of their facilities in terms of maintenance and monitoring. In conclusion, we raise the following points:

• STL specifications can help track the behaviour over time of WRRFs and identify *via* effluent concentrations if there is a lack of resilience in the facility in order to plan interventions. The STL specification describes the compliance requirements using an easy to check logical syntax.

• Resilience is a system specific metric, so failure modes of a wastewater facility are an intrinsic characteristic of that system. Resilience analysis for specific threats can help identify the resilience threshold values in order to avoid compliance failure.

• The recovery time *T* after a failure is not set by the water companies. If set, it can help the water companies better understand the resilience of their facilities.

• This framework enables water companies to better monitor their WRRF's resilience by improving how water companies check compliance using the data that they routinely and regularly collect for facilities under their management.

• Analysis of the robustness of the WRRF can help the water companies understand how the system is operating, in terms of meeting compliance, under normal operating conditions. Then, a comprehensive study of stressors affecting the WRRF can help identify potential vulnerabilities.

• A real-time controller in Supervisory Control and Data Acquisition (SCADA) systems with implemented STL specifications can enhance the monitoring of WRRFs leading to better resource management. Resilient processes lead to more reliable facilities that enable the recovery of more nutrients, energy, and other resources, while recycling water safely to the environment.

The proposed metric provides a unified way of assessing resilience quantitatively. It will also be possible to use the resilience values for comparing resilience of different plants. For instance, water companies invest more in monitoring and maintenance of bigger WRRFs. It will allow hypothesis testing for general resilience of bigger WRRFs compared with smaller ones. Furthermore, their redundancies are generally higher; having spare components to overcome failures in case of unexpected threats. Small WRRFs are sampled less frequently and, as a consequence, if a failure happened it is impossible to estimate the recovery time just by looking at samples taken at specific time point. Therefore, real-time monitoring of resilience embedded in the SCADA system, or in a digital twin of a facility, could help water companies visualise if the WRRF meets the STL specification, and so prioritise interventions that enhance the resilience of their WRFFs.

## 6 Future development

As future developments, we aim to implement the whole logic framework of Fig. 1 in order to add further complexity. The above computations can be extended to include random duration and random starting times of the stressor as considered in ref. 48. Also, a different design storm could be used for the computation of the resilience of the system under rainfall events. Probabilistic analysis can lead to a heat-map graph considering the return period of different scenarios linked to climate change or population growth. For water companies, it is particularly useful to build a heat map with the return period of different scenarios. Furthermore, future developments aim to classify modes of failure in a WRRF. This will help with making design decisions that avoid catastrophic failures, while also being more tolerant to minor failures, reducing costs. It may be possible to discriminate between failures with the same expected damage/utility by being more averse toward catastrophic failures (lower probability but higher impact), and being more tolerant toward recurring interruptions (high probability, low impact failures). The analysis of the resilience of the system can be applied to specific days of testing, *e.g.* the 52nd day, which indicated by the least robustness in Fig. 6.

## Code availability

The python codes can be found at the following link: https://github.com/annalaino/coding-paper.git.

## Data availability

The data and Python code were developed in collaboration with industrial partners and cannot be published online due to a non-disclosure agreement (NDA) between the authors and the industrial partners. A simplified version of the code may be published in the future, pending approval from the industrial partners. If approved, a link to the code will be included in the final version of the paper.

## Conflicts of interest

There are no conflicts to declare.

## Figures and Tables

**Fig. 11:**
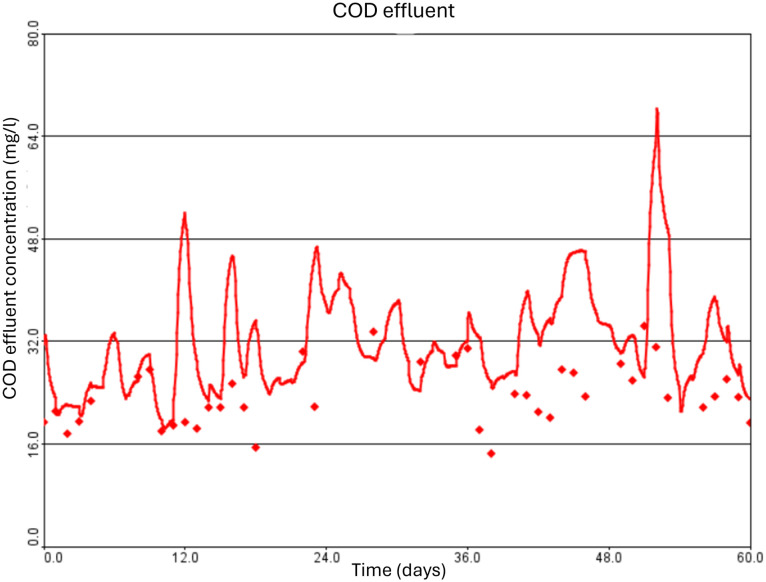
Effluent concentrations of COD effluent concentration (mg l^−1^). The continuous red line is the modelled COD effluent concentration and the red diamonds are the measured composite data.

**Fig. 12:**
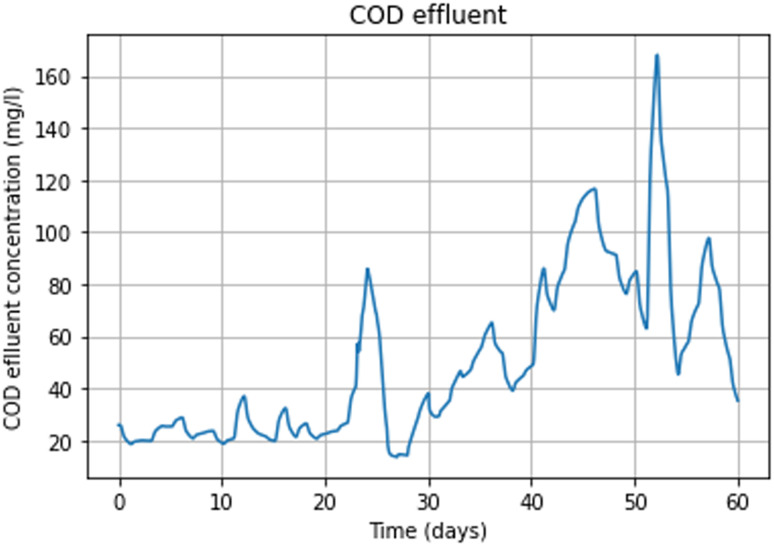
COD effluent concentration (mg l^−1^) of one of the Monte Carlo simulations in GPS-X.

## Appendices

### Appendices

#### A Signal temporal logic specifications

We consider the state of a system in an infinite trajectory as *ξ* = *x*(0)*x*(1)*x*(2)… where *x*(*t*) is the state of the system at a specific time *t* ∈ ℕ: = {0, 1, 2, …}.

##### Definition 3 syntax

A signal temporal logic formula is defined using the following syntax:where T is the true predicate, F is the false predicate, and *μ*: ℝ → {T, F} is a predicate where the sign of the function of the state determines its truth value, *i.e. μ*(*x*) = T if and only if *α*(*x*) ≥ 0 with *α*: ℝ*n* → ℝ considered to be an affine function of the state and associated with *μ*. The operators  and ∧ are respectively negation and conjunction. *U*_[*a*,*b*]_ is the until operator with *a*, *b* ∈ ℝ ≥ 0.

##### Semantics

Below the satisfaction of an STL formula by a trajectory *ξ* is defined recursively: such that  and for all .

A trajectory *ξ* satisfies a specification *ψ*, denoted by , if . Moreover, other operators can be defined as follows:

• disjunction:

• eventually:

• always:

The horizon of an STL formula is defined by the len(*ψ*) which the maximum threshold value of an interval, which is also the length of the interval where the satisfaction of  is studied.

#### B Robustness of STL specifications

The robustness of the formula *ψ* is defined as a real valued function *ρ*^*ψ*^ assigned to an STL formula *ψ*. Note that *ρ*^*ψ*^(*ξ*, *t*) > 0 implies that . Robustness of an STL formula is computed recursively according to the structure of the formula as follows:*ρ*^T^(*ξ*, *t*) = + ∞,*ρ*^*μ*^(*ξ*, *t*) = *α*(*ξ*(*t*)) where *μ*(*ξ*(*t*)) = T if *α*(*ξ*(*t*)) ≥ 0,*ρ*^*ψ*∧*ϕ*^(*ξ*, *t*) = min(*ρ*^*ψ*^(*ξ*, *t*), *ρ*^*ϕ*^(*ξ*, *t*)),The STL robustness and satisfaction are defined in relation with the trajectory of the system. If the system is stochastic, the trajectory is a stochastic process, and as a consequence the satisfaction relation is a Bernoulli random variable and the robustness is a real random variable.

#### C COD calibration in GPS-X

Fig. 11 shows the COD effluent concentration (mg l^−1^) modelled in GPS-X and compared with measured data. The continuous red line represents the COD concentrations predicted by the model, while the red diamonds denote the actual measured data points. This visual comparison allows for an assessment of how well the model tracks the real-world measurements over time.

Fig. 12 shows the COD effluent during one of the Monte Carlo simulations under a stressor.

#### D Linear mapping

Eqn (1) shows the linear mapping function applied to the Rob(*ψ*). We determined the upper and lower bounds by selecting the maximum and minimum values between the BOD influent and BOD effluent. The same methodology was used for COD. Typically, the maximum value for both BOD and COD corresponds to the influent, while the minimum value corresponds to the effluent. The linear mapping formulas are

where BOD_i_(*t*) and BOD_e_(*t*) respectively represent the influent and effluent concentration of BOD_5_. Similarly, COD_i_(*t*) and COD_e_(*t*) denote the influent and effluent concentration of COD. We also applied the linear mapping for the upper and lower tier of the compliance requirements.• BOD_UT_ = 50, BOD_LT_ = 25;• COD_UT_ = 250, COD_LT_ = 125.

Then the linear mapping is applied to upper tier and lower tier for BOD and COD:• % reduction_BOD_ = 0.7• % reduction_COD_ = 0.75.The percentage of reduction for BOD and COD were already in the expected range. Furthermore, we have for the *c* computation:• UT_BOD_(*t*) = UTnorm_BOD_ − linBOD_e_(*t*)• LT_BOD_(*t*) = LTnorm_BOD_ − linBOD_e_(*t*)• BOD_p_(*t*) = −(% reduction_BOD_ × linBOD_i_(*t*)) + linBOD_i_(*t*) − linBOD_e_(*t*)• max1(*t*) = maximum(LT_BOD(*t*)_, BOD_p_(*t*))• *c*_BOD_(*t*) = minimum(max1(*t*), UT_BOD_(*t*))• UT_COD_(*t*) = UTnorm_COD_ − linCOD_*e*_(*t*)• LT_COD_(*t*) = LTnorm_COD_ − linCOD_e_(*t*)• COD_p_(*t*) = −(% reduction_COD_(*t*) × linCOD_i_(*t*)) + linCOD_i_(*t*) − linCOD_e_(*t*)• max2 = maximum(LT_COD_(*t*), COD_p_(*t*))• *c*_COD_(*t*) = minimum(max2(*t*), UT_COD_(*t*))• *c*(*t*) = minimum(*c*_BOD_(*t*), *c*_COD_(*t*)).
